# Genetic C3 variants reveal functional independence between extracellular and intracellular forms of complement C3

**DOI:** 10.1007/s00018-026-06517-2

**Published:** 2026-11-03

**Authors:** Daniel Chacon-Arguedas, Ben C. King, Ana V. Marin, Agustin Tortajada, Gregers Rom Andersen, Jose R. Regueiro, Anna M. Blom

**Affiliations:** 1 Department of Translational Medicine, Lund University, Malmö, Sweden; 2 Department of Immunology, Ophthalmology and ENT, Complutense University School of Medicine and 12 de Octubre Health Research Institute (imas12), Madrid, Spain; 3 Department of Molecular Biology and Genetics, Aarhus University, Aarhus, Denmark

**Keywords:** Complement, C3, Gene variant, Intracellular complement, C3 deficiency.

## Abstract

Complement component C3 is the central effector of extracellular complement activation, but recent studies have identified intracellular forms of C3 with distinct non-canonical functions. How pathogenic C3 variants affect intracellular C3 biology remains unknown. Here, we investigated two previously described C3 deficiency-associated variants, c.168_169del and c.1682G > A (p.G561D), linked to severe plasma C3 depletion. Unexpectedly, both variants remained detectable intracellularly despite markedly reduced extracellular C3 levels observed in the patients. The c.168_169del frameshift variant, predicted to abolish protein expression, generated a smaller non-secreted pro-C3 species lacking glycosylation and localized exclusively to the cytosol. Cellular fractionation, antigenic characterization, and split-GFP localization assays using cell models demonstrated that this protein resembles a non-canonical cytosolic C3 isoform generated through alternative translation initiation downstream of the mutation site. These findings provide clinical support for the existence of cytosol-restricted C3 generated independently of the canonical secretory pathway. In contrast, the p.G561D variant displayed normal intracellular expression, antigenicity, glycosylation, and secretion but showed impaired alternative pathway convertase activity, increased susceptibility to factor H/factor I-mediated degradation, and enhanced spontaneous turnover in serum, explaining the severe plasma C3 depletion observed in the patient. Collectively, our results demonstrate that pathogenic C3 variants can exert fundamentally different effects on extracellular and intracellular C3 pools. These findings support the concept that intracellular and extracellular C3 represent functionally independent compartments and highlight the importance of integrating intracellular complement biology into the interpretation of complement-associated diseases.

**Supplementary Information:**

The online version contains supplementary material available at https://doi.org/10.1007/s00018-026-06517-2.

## Introduction

The complement system, traditionally associated with the humoral arm of innate immunity, comprises more than 50 fluid-phase and membrane-bound proteins and peptides widely distributed in plasma and tissues [1–3]. Complement activation is classically associated with three major functional outcomes: modulation of the inflammatory response, opsonization of surfaces and lysis of microorganisms due to MAC assembly [1–4]. Complement activity is tightly regulated by a group of inhibitors that control cascade activation through decay acceleration of convertases, proteolytic inactivation, or competitive inhibition [1, 2, 4]. Beyond these canonical functions, growing evidence supports the involvement of complement in previously unrecognized processes, including regulation of adaptive immunity [5, 6], promotion of tissue regeneration and angiogenesis [7], mobilization of stem cells [8, 9], homeostasis of the central nervous system [10, 11], and metabolism [12, 13].

Complement component 3 (C3) is the central molecule of the complement cascade [14]. Most of the circulating plasma C3 is produced by hepatocytes in the liver; however, C3 is expressed by many cell types in the human body. The distribution of the protein is also extensive, present in plasma, tissues and intracellular compartments. These diverse niches reflect a complex set of different activation states and functions [14]. The study of intracellular C3 has revealed a growing number of non-canonical roles that differ markedly from its classical extracellular functions [15–18].

Two sources of cytosolic C3 have recently been described. The first corresponds to canonical mature C3, normally destined for secretion, that undergoes retrotranslocation from the endoplasmic reticulum (ER) into the cytosol. The second, a hypothesized source of cytosolic pro-C3 is a non-canonical C3 isoform generated through alternative translation initiation at downstream start codons. Two alternative in-frame translation initiation codons (M25 and M42) have been previously identified and validated by our group. Interestingly, when all three initiation codons were mutated, cytosolic C3 remained detectable, suggesting the possible existence of additional alternative translation initiation sites [19]. This intracellular isoform differs substantially from canonical C3: it is synthesized directly in the cytosol, is non-glycosylated, is not proteolytically processed into α- and β-chains, and the reducing intracellular environment likely influences its redox state and disulfide bond formation [19]. This is supported by functional evidence from gene-edited cell lines only able to express this form of intracellular C3, but not secreted C3 [20, 21].

Intracellular C3 has been implicated in several non-canonical functions, primarily related to cellular metabolism and homeostasis, including regulation of gene transcription [22], autophagy [23], lipid metabolism [24], glycolysis [25], cytokine production [21], cell proliferation and survival, and mitochondrial respiration [15]. This functional divergence between extracellular and intracellular C3 supports the concept of C3 compartmentalization, whereby distinct molecular forms fulfill specialized roles depending on their cellular localization [16].

Given its central role in complement function, C3 deficiency leads to severe clinical consequences. Primary C3 deficiency is a rare genetic disorder, with fewer than 30 cases reported worldwide [26, 27]. Affected individuals typically present with recurrent bacterial infections, particularly caused by Gram-negative bacteria such as *Neisseria meningitidis*, as well as *Streptococcus pneumoniae* and group A *Streptococcus* [26]. In some cases, immune complex–mediated diseases, including renal or rheumatologic manifestations, may also develop [26]. Although the molecular basis of reported cases has been characterized, little is known about the impact of C3 deficiency on intracellular C3 isoforms.

**Fig. 1:**
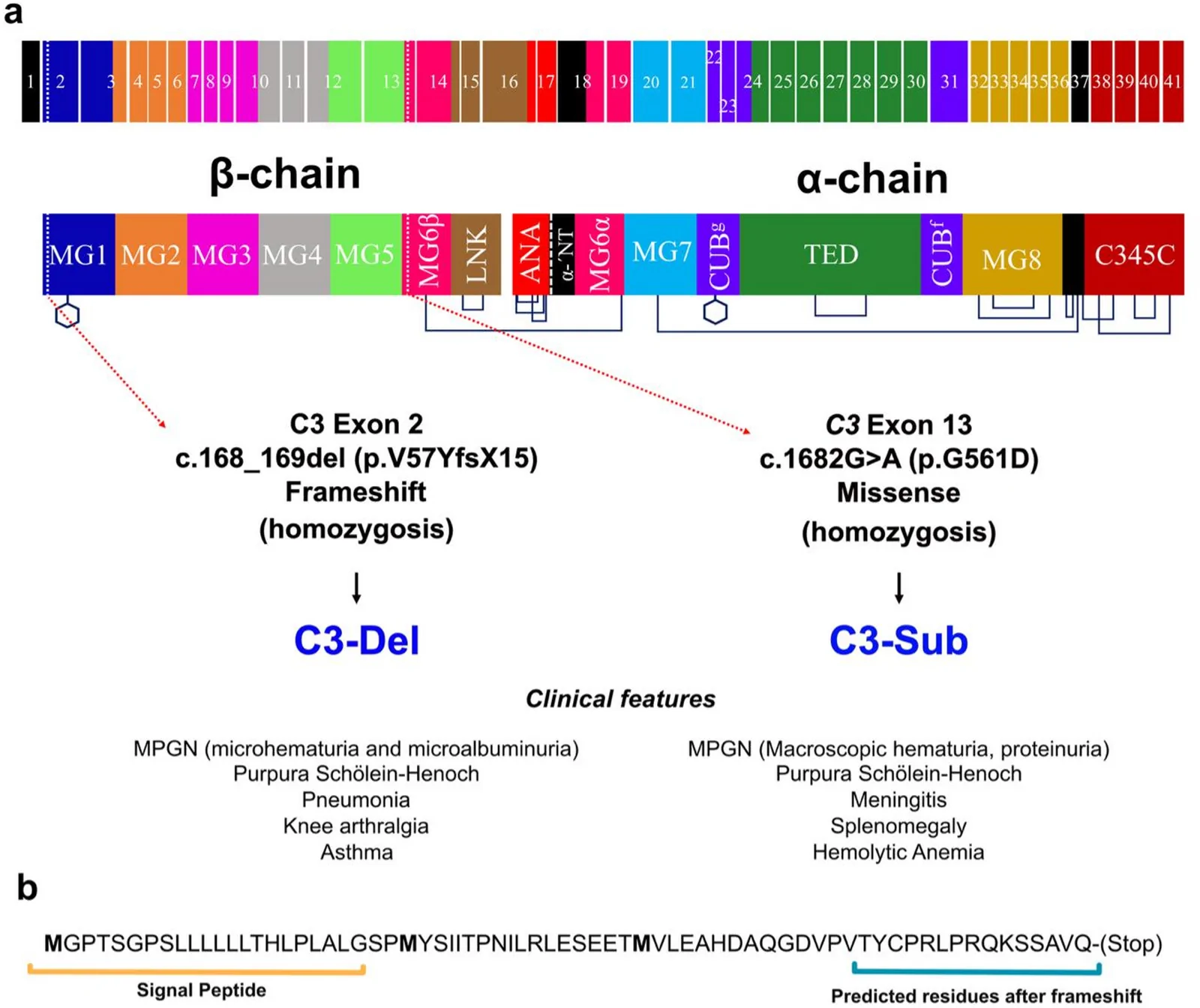
Summary of the C3 variants studied in this work. (**a**) The location of the mutations within the mRNA exons (above) as well as within the mature protein domains (below) are marked by the dotted lines. In addition, the gene defect and effect on this protein are stated, as well as the main clinical features exhibited by the patients with these variants. In the protein scheme, disulfide bond positions are shown by black lines and N-glycosylation sites by hexagons. (**b**) Predicted truncated product produced after translation of *C3* c.168_169del C3-Del mutant. The translation initiation methionines are shown in bold: the first one mediates the synthesis of canonical, secreted C3, whereas the two downstream alternative initiation start sites are responsible for the synthesis of cytosol-exclusive forms of C3

The genetic variants in *C3* studied in this work (Fig. 1) were previously reported together with detailed clinical, genealogical, and molecular characterization of the affected patients [6]. Both variants were associated with severe plasma C3 depletion and were classified as loss-of-function mutations, leading to the diagnosis of primary C3 deficiency.

Here, we investigated the effects of these mutations on intracellular C3 forms. We demonstrate that these C3 variants can be expressed intracellularly, even when secretion is severely impaired for one of them. Notably, we show that cells still express a C3 variant from a mutation predicted to completely abolish protein synthesis, likely through alternative translation mechanisms that generate a cytosol-restricted C3 isoform. This clinical example provides validation of the model of cytosolic C3 expression by alternative translation. Furthermore, our findings highlight the relevance of analyzing intracellular C3 forms for functional variant characterization and diagnostic interpretation. Collectively, these results demonstrate that pathogenic genetic variants can have distinct effects on intracellular and extracellular compartment C3, and that intracellular C3 expression may persist despite severe impairment of C3 secretion or plasma availability.

## Materials and methods

### Cell culture

HEK293 and HepG2 cells were cultured in Dulbecco’s Modified Eagle Medium (DMEM, SH30243.1; HyClone) supplemented with 10% heat-inactivated Fetal Bovine Serum (SV30160.02, HyClone), 100 units/mL of Penicillin/ 100 µg/mL Streptomycin (SV30010; HyClone) and 1X L-Glutamine (A29168-01; Gibco). Cells were maintained at 37 °C in a humidified atmosphere containing 5% CO₂ and they were routinely tested for mycoplasma contamination (Eurofins Genomics) and confirmed negative.

### Plasmids and site‑directed mutagenesis

Full-length human C3 cDNA was cloned into pcDNA3 (Invitrogen). Mutations were introduced using the QuikChange II Site-Directed Mutagenesis Kit (Agilent Technologies) according to the manufacturer’s instructions. Primers used were as follows: C3-Del (Forward: 5′-GGGATGTTCCAGTCACTTACTGTCCACGACTTCCC-3′ and Reverse: 5′-GGGAAGTCGTGGACAGTAAGTGACTGGAACATCCC-3′); C3-Sub (Forward: 5′-GACTCCTGCGTGGACTCGCTGGTGGTAAAAAGCGG-3′ and Reverse: 5′-CCGCTTTTTACCACCAGCGAGTCCACGCAGGAGTC-3′). For constructs carrying mutations in ATG1, ATG2, and ATG3, site-directed mutagenesis was performed as previously described [19]. For constructs with mutations in ATG1-4 (M1I, M25I, M42I, M83I) and ATG1-5 (M1I, M25I, M42I, M83I, M164I), synthetic DNA fragments flanked by HindIII and Bpu1102I restriction sites were purchased from GeneArt (Thermo Fisher Scientific) and cloned into the C3-WT vector. All constructs were verified by Sanger sequencing and/or whole-plasmid sequencing (Eurofins Genomics).

### Cell transfection

HEK293 cells (2 × 10⁵ cells/well) or HepG2 C3 knockout (KO) cells (1 × 10⁵ cells/well) were seeded in 24-well plates in complete DMEM. After 24 h, medium was replaced with Opti-MEM^®^ I + GlutaMAX™ (51985-026, Gibco). Cells were transiently transfected with C3 expression plasmids using Lipofectamine 3000 (Invitrogen) according to the manufacturer’s instructions.

After 24 h, supernatants and cells were collected. Cells were lysed in ice-cold RIPA buffer (50 mM Tris-HCl, pH 7.4; 150 mM NaCl; 1% NP-40; 0.5% sodium deoxycholate) supplemented with Halt™ protease and phosphatase inhibitor cocktail (78440, Thermo Scientific). Lysates were incubated on ice for 30 min with intermittent vortexing and clarified by centrifugation at 15,000 × g for 10 min at 4 °C.

### Cell fractionation

HepG2 C3 KO cells were transiently transfected with C3 expression plasmids using Lipofectamine 3000 (Invitrogen) according to the manufacturer’s instructions. After 24 h, cells were harvested, and cytoplasmic and membrane/organelle fractions were isolated using the Mem-PER Plus Membrane Protein Extraction Kit (89842, Thermo Scientific) according to the manufacturer’s instructions, with minor modifications. Cell pellets were resuspended in 500 µL permeabilization buffer I supplemented with Protease Inhibitor Cocktail and incubated for 10 min at 4 °C. The lysates were subsequently centrifuged at 16,000 x g for 10 min, and the supernatants containing cytoplasmic proteins were collected and stored at - 20 °C until further use. After washing with PBS, the pellets were resuspended in 500 µL solubilization buffer II supplemented with Protease Inhibitor Cocktail and incubated for 30 min at 4 °C. The lysates were then centrifuged at 16,000 x *g* for 15 min, and supernatants or membrane fractions were collected and stored at -20 °C until further use.

### Immunoprecipitation of C3 from cell lysates

Clarified lysates were pre-cleared with Dynabeads™ Protein G magnetic beads (10003D; Invitrogen) for 1 h at 4 °C. Anti-C3c antibody (10 µg; A0062, Agilent Dako) was pre-bound to magnetic beads incubated with lysates overnight at 4 °C. Beads were washed three times with SDS-free RIPA buffer. Bound proteins were eluted by boiling the samples at 95 °C for 5 min in Lane Marker Reducing Sample Buffer (39000; Thermo Scientific).

### SDS-PAGE and western blotting

Supernatants, fractions or immunoprecipitated samples were separated by SDS-PAGE under reducing or non-reducing conditions as indicated and transferred to PVDF membranes using a semi-dry blotting apparatus (BioRad). Membranes were blocked for 1 h at room temperature (RT) in Quench Buffer (50 mM Tris–HCl pH 8.0, 150 mM NaCl, 0.1% Tween 20, 3% fish gelatin, Sigma Aldrich) and then incubated with the primary antibodies overnight at 4 °C. For C3 detection, the following antibodies were used: Goat polyclonal anti-human C3 antibodies (A304, Quidel and 204869, Calbiochem), Mouse monoclonal anti-C3/C3a (HM2073, HyCult), Mouse monoclonal Anti-C3b/iC3b/C3c antibody (recognizing C3 β-chain) [28], Rabbit monoclonal anti-C3 (termed anti-C3 C-term; ab11871, Abcam). As loading controls for cell fractions, rabbit monoclonal anti-β tubulin (Ab6046, Abcam) or mouse monoclonal anti-mammalian protein disulphide-isomerase (PDI) antibody (ADI-SPA-891-D, Enzo Life Sciences) were used. Proteins were visualized using polyclonal HRP-conjugated antibodies against rabbit, mouse, or goat immunoglobulin (Dako) diluted in Quench buffer. For glycosylation analysis, membranes were incubated with biotinylated concanavalin A (C2272, Sigma Aldrich; 5 µg/mL in Quench) followed by HRP-conjugated Streptavidin (S911, Invitrogen). After incubation with primary and secondary antibodies, the membranes were washed three times with Immunowash buffer (50 mM Tris–HCl (pH 8.0), 150 mM NaCl, 0.1% Tween 20). Membranes were developed by the enhanced chemiluminescence (ECL) method (Millipore), and pictures were captured with the ChemiDoc MP system using Image Lab software from Bio-Rad.

### C3 ELISA

C3 concentration in the supernatants or lysates was measured by sandwich ELISA. Maxisorp microtiter 96-well plates (Invitrogen) were coated overnight at 4 °C with rabbit anti-human C3c (A0062, Dako; 1:1000 in PBS). Plates were washed four times between steps with Immunowash buffer and blocked for 1 h at 37 °C with Quench buffer, then incubated with 50 µl/well of samples or purified C3 (A113, Complement Technologies) standard (range: 500 − 7.8 ng/ml). Bound proteins were detected using goat anti-human C3 antibody (A304, Quidel; 1:5000 in Quench) and HRP-conjugated rabbit anti-goat immunoglobulins (Igs) (P0049, Dako; 1/2000 in Quench​) for 1 h at RT. TMB substrate (Kementec) was used for detection; the reaction was stopped with 0.5 M sulphuric acid, and absorbance was measured at 450 nm.

### HEK293-sfGFP_1−10_ reporter cell line

HEK293 cells (2 × 10⁵ cells/well) were co-transfected with a PiggyBac plasmid encoding sfGFP_1−10_ and pcDNA3.1(+)-hyPBase at a cargo-to-transposase ratio of 5:1 using Lipofectamine 3000 (Invitrogen) according to the manufacturer’s instructions. After 72 h, the selection of cells that had successfully integrated the cargo into their genome was initiated by culturing the cells in DMEM supplemented with 1 µg/mL puromycin. The medium was replaced every other day until all cells in the control population had died.

### Split-GFP based localization assay

HEK293-sfGFP_1− 10_ were transiently transfected with pcDNA3.1(+) plasmids containing C3-WT, C3-Del and C3-ΔATG1 (cytosolic C3, used as control) fused to the small GFP_11_ fragment. After 24 h, cells were analyzed by confocal microscopy and flow cytometry. For confocal imaging, cells were fixed with 4% paraformaldehyde in 1X PBS, treated with 50 mM NH_4_Cl, stained with Hoechst 33,342 (H3570, Molecular Probes), and mounted using ProLong™ Glass Antifade Mountant (P36980, Invitrogen). Images were acquired using a Zeiss LSM 800 confocal microscope equipped with Airyscan and operated with ZEN (blue edition) software. For flow cytometry analysis, cells were harvested, washed, and stained for viability with BD Horizon™ Fixable Viability Stain 780 (565388, BD Biosciences). Samples were analyzed using a CytoFLEX cytometer (Becton Dickinson).

### Expression of recombinant C3 variants

C3-Sub and wild-type C3 encoding plasmids were transiently transfected into Freestyle 293-F cells (Thermo Fisher Scientific) using FreeStyle MAX reagent (Thermo Fisher Scientific) according to the instructions of the manufacturer. Supernatants were collected on day 5 post-transfection and concentrated using Amicon^®^ Ultra Centrifugal Filter, 100 kDa MWCO (UFC9100, Millipore). Supernatants and concentrated fractions were kept at 4 °C throughout all the steps. C3 concentrations were quantified by ELISA as described above and used for functional assays.

### Alternative pathway C3-convertase assembly assay

Fluid-phase alternative pathway (AP) C3 convertase activity was assessed by measuring C3 proteolysis and factor B (FB) cleavage. Each reaction contained 250 ng C3 variant or control C3, 250 ng FB (A135, Complement Technologies), and 7 ng factor D (FD; A136, Complement Technologies) in Mg²⁺-EGTA buffer (2.5 mM veronal buffer, pH 7.3; 70 mM NaCl; 140 mM glucose; 0.1% gelatin; 7 mM MgCl₂; 10 mM EGTA). Reactions were incubated for 30 min at 37 °C and stopped with SDS sample buffer. Samples were heated at 95 °C for 5 min and analyzed by SDS-PAGE (4–20% gradient gels), followed by Western blotting (as explained above). Intact factor B and the cleavage product Bb (60 kDa) were then detected using a polyclonal goat anti-human factor B antibody (A235, CompTech). C3 and C3b were detected using a goat polyclonal anti-human C3 antibodies (204869, Calbiochem), followed by an HRP-conjugated rabbit anti-goat antibody. Purified C3 (A113, Complement Technologies) was used as a positive control. The blots were developed using enhanced chemiluminescence (Merck-Millipore). Band intensities (intact FB vs. Bb; C3 α-chain vs. C3b α′-chain) were quantified by densitometry and expressed as ratios.

### Degradation of C3 by factor I

The C3 activation product C3b and the C3 thioester hydrolysis product C3(H_2_O) can be degraded by factor I (FI), in the presence of cofactors such as factor H (FH). A mixture containing 250 ng of C3-WT, C3-Sub, or C3 treated with methylamine (C3-Met, a C3(H_2_O) proxy), 300 ng of FH (A137, CompTech) and 50 ng of FI (A138, CompTech) was prepared in Mg^2+^ EGTA buffer. Next, samples were then incubated for 30 min, and the reaction was terminated by the addition of SDS-PAGE sample buffer, containing 25 mM DTT. The samples were heated at 95 °C for 5 min and analyzed by SDS-PAGE (4–20% gradient gels), and Western Blotting as described above. C3-Met served as a positive control. The ratio of intact C3 α-chain to the 43-kDa degradation fragment was determined by densitometric analysis.

### Degradation of C3 in C3-depleted serum

To evaluate the stability and spontaneous hydrolysis of C3-Sub, 250 ng of C3-WT or C3-Sub were incubated in 2% C3-depleted serum (A314, CompTech) prepared in Mg^2+^ EGTA buffer. Samples were incubated at 37 °C for 30 min, 2 h, or 4 h. Reactions were terminated by the addition of SDS-PAGE sample buffer, containing 25 mM DTT. Samples were heated at 95 °C for 5 min and analyzed by SDS-PAGE using 4–15% gradient gels followed by Western Blotting, as described above, using a Rabbit Polyclonal serum anti-C3 α-chain (ab97462, Abcam). The ratio of intact C3 α-chain to the 43-kDa degradation fragment was determined by densitometric analysis.

### ELISA for C3(H_2_O)

To evaluate whether C3-Sub adopts a C3(H_2_O)-like conformation, an ELISA protocol described previously was used [29]. Briefly, MaxiSorp 96-well microtiter plates (Invitrogen) were coated overnight at 4 °C with anti-C3b/iC3b monoclonal antibody clone 3E7 (HM2286, HyCult). Plates were washed four times with Immunowash buffer, blocked for 1 h at room temperature (RT) with Quench buffer, and subsequently incubated with 50 µl/well of samples, purified C3 or methylamine-treated C3 standards (4–500 ng/mL). Bound proteins were detected using rabbit anti-human C3a polyclonal serum (A218, CompTech) followed by HRP-conjugated goat anti-rabbit immunoglobulins (P0448, Dako) for 1 h at RT. TMB substrate (Kementec) was used for detection; the reaction was stopped with 0.5 M sulphuric acid, and absorbance was measured at 450 nm.

### Molecular modelling

The crystal structures of the alternative pathway C3 convertase C3bBb in complex with C3 (PDB ID: 9U62) and C3b in complex with FH CCP1-4 (PDB ID: 2WII) were obtained from the RCSB Protein Data Bank (https://www.rcsb.org/). Molecular graphics and analyses were performed with UCSF ChimeraX (UCSF/NIH) [30] and PyMol (The PyMOL Molecular Graphics System, Version 3.0 Schrödinger, LLC).

### Data and statistical analysis

Western blot band intensities were quantified using Image Lab™ (Bio-Rad). Graphs and statistical analyses were done using GraphPad Prism v10.6.0 (GraphPad Software). Normality was assessed prior to parametric testing. For normally distributed data, paired two-tailed t tests or one-way ANOVA were applied as appropriate. If normality was not met, data transformation was attempted; if unsuccessful, nonparametric tests were used. Data are presented as mean ± SD unless otherwise indicated. Statistical significance was defined as follows: ns *p* ≥ 0.05; *p = 0.01 − 0.05; **p = 0.001 − 0.0​1; *** *p* = 0.0001 − 0.001; ****p = < 0.0001.

## Results

### Plasma C3-deficiency causing variants do not prevent C3 expression and/or secretion in cell lines

Patients carrying studied variants exhibited a severe plasma deficiency of C3, attributed to frameshift or missense alterations in the C3-coding sequence, as predicted by in-silico analyses. However, no biological assays had been performed to experimentally confirm these predictions [6]. Considering recent reports describing novel intracellular functions and non-canonical roles of C3, we sought to investigate the intracellular fate of these C3 variants. The G561D substitution in the MG6 domain was termed C3-Sub, while the double-nucleotide deletion causing a frameshift in the MG1 domain was termed C3-Del (Fig. 1).

**Fig. 2:**
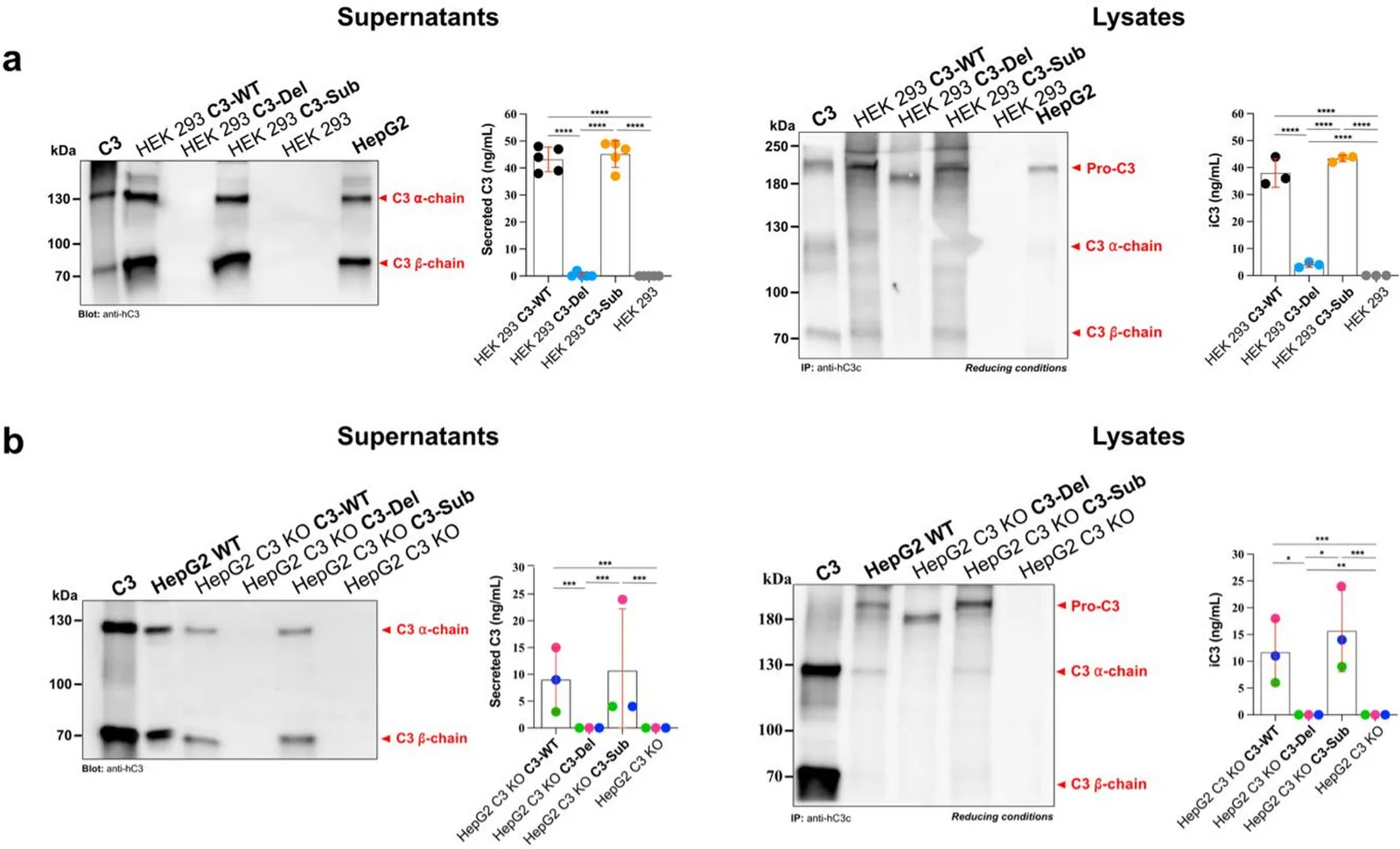
C3-Del variant produces a shorter and non-secreted pro-C3, while C3-Sub secretion and intracellular pro-C3 are comparable to the wild-type C3. HEK 293 cells (**a**) and three different HepG2 C3 Knockout cell clones (**b**) were transfected with pcDNA3 plasmids containing wild-type or the pathogenic variants of C3 (C3-Del and C3-Sub), and 24 h later the supernatant and whole-cell lysates were collected. The expression of C3 was analyzed by western blot (for the lysates, preceded by an immunoprecipitation of C3). Overexposed blots showed comparable results. The concentration of secreted and cytosolic C3 was measured by ELISA. Data are presented as mean ± SD from 5 independent experiments. Statistical significance was determined using One-way ANOVA. ns *p* ≥ 0.05; *p = 0.01 − 0.05; **p = 0,​001 − 0,​01; *** *p* = 0.0​001 − 0.0001; ****p = < 0.0001

First, the synthesis and secretion of the protein variants from cloned cDNA were examined in HEK 293 cells, a cell line that does not express endogenous C3. The levels of secreted C3-Sub were comparable to those of wild-type C3 (C3-WT), and no differences in the apparent molecular weight of the α and β chains were detected (Fig. 2a, left). We next assessed the synthesis of C3 by measuring the content of intracellular C3. In cells transfected with C3-WT, the predominant intracellular form was proC3 with low levels of mature forms of α- and β-chains (Fig. 2a, right). A similar pattern was observed in cells expressing C3-Sub, with no significant differences in the molecular weight or abundance of both mature and proC3 compared with C3-WT-expressing cells.

In contrast, no secreted C3 was detected in the supernatant of cells transfected with the C3-Del variant after 24 h, as assessed by immunoblotting or ELISA. Unexpectedly, pro-C3 was detected in the lysates of C3-Del-expressing cells; this proC3-Del protein exhibited an apparent molecular weight approximately 10 kDa lower than that of proC3-WT. Overall protein levels were markedly reduced, and no processed mature α- or β-chains were detected (Fig. 2a, right).

Given the high transfection efficiency and robust recombinant protein production in HEK 293 cells, we sought to validate these findings in a more physiologically relevant model. To this end, we used HepG2 cells, a hepatocarcinoma-derived cell line that endogenously synthesizes and secretes C3. Several C3-knockout HepG2 clones were transfected with plasmids encoding the different C3 variants. The results in HepG2 cells recapitulated those obtained in HEK 293 cells. C3-Sub was synthesized and secreted at levels comparable to C3-WT. In contrast, no secretion of C3-Del was detected, although the truncated pro-C3 form was observed in cell lysates by immunoblotting (Fig. 2b).

Taken together, these results indicate that both C3 variants are sufficiently stable within the intracellular compartment to be detected by conventional assays. Moreover, the normal secretion profile of C3-Sub relative to C3-WT in cell lines calls into question the primary deficiency diagnosis of the patient, who exhibited markedly reduced plasma C3 levels.

### Antigenic characterization demonstrated that both C3 variants are similar to wild-type C3 in cell lines

**Fig. 3:**
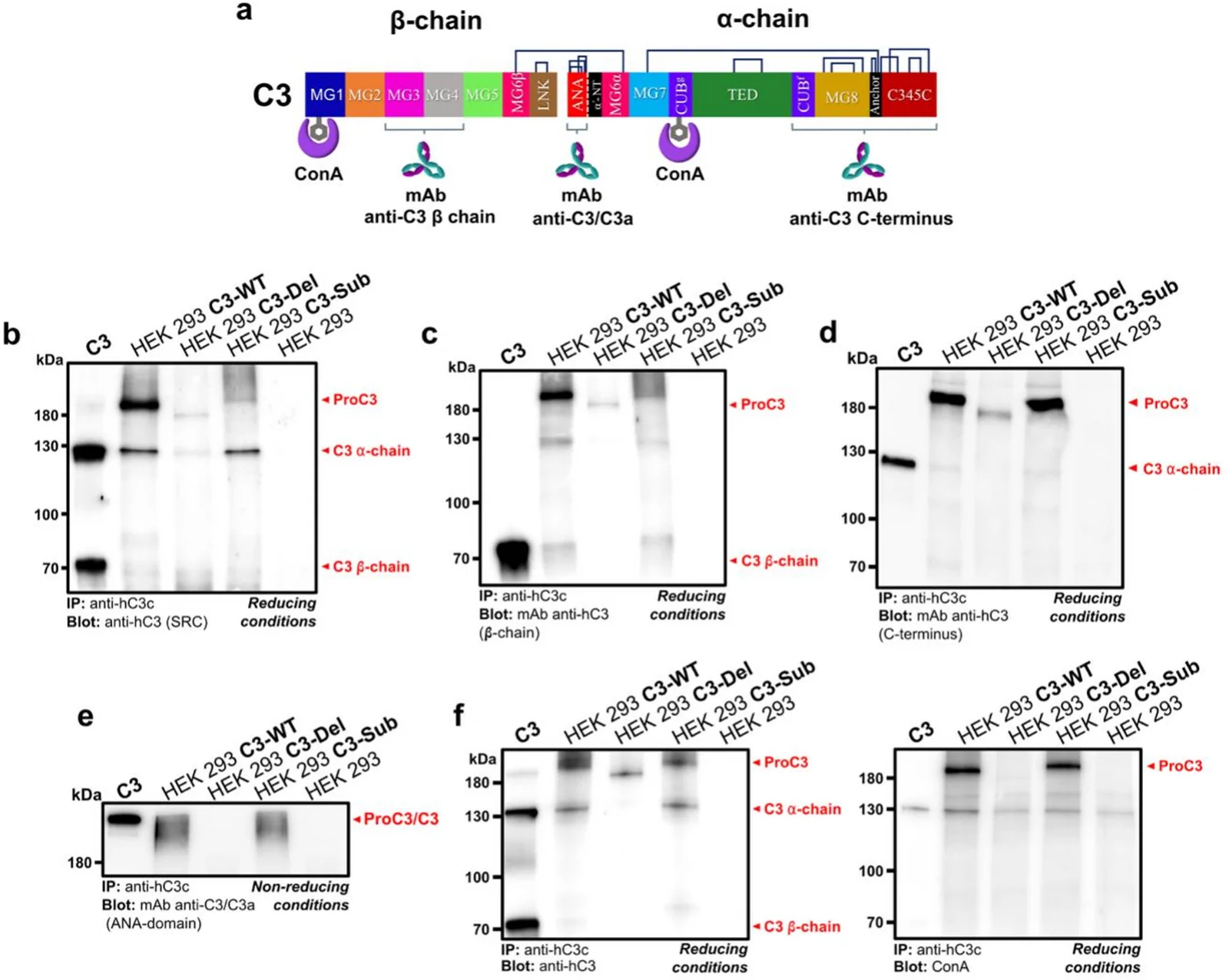
Antigenic determinant mapping of C3 variants in whole cell lysates. HEK293 cells were transfected with pcDNA3 plasmids encoding C3-WT or the pathogenic variants C3-Del and C3-Sub. Twenty-four hours post-transfection, whole-cell lysates were collected, and C3 was immunoprecipitated. The precipitated pro-C3 was analyzed by immunoblotting for different antigenic determinants (**a**) using a different in-house produced polyclonal serum (**b**) and monoclonal antibodies recognizing distinct epitopes: β-chain (**c**), C345C domain (**d**), C3a/ANA domain (**e**), and glycosylation sites (**f**). The data indicate that the C3-Del variant produces a shorter, non-proteolytically processed and non-glycosylated pro-C3, whereas C3-Sub is indistinguishable from wild-type C3 (C3-WT). Data are representative of at least three independent experiments (*n* ≥ 3)

The *C3* c.168_169del C3-Del mutation introduces a frameshift predicted to result in premature termination by a stop codon [6]. Nonetheless, our data revealed the presence of a smaller proC3 species that was still recognized in Western Blotting by a polyclonal anti-C3 serum (Fig. 2). To confirm that the band observed in immunoblots corresponded to proC3, we performed epitope mapping using a panel of antibodies and lectins recognizing distinct C3 domains or post-translational modifications (Fig. 3a). The C3-Sub variant was included as an internal control, as the predicted amino acid substitution resulting from the c.1682G > A mutation is not expected to affect recognition of the selected epitopes, particularly under non-reducing conditions.

As an initial validation, we employed an independent polyclonal rabbit anti-human C3 serum (kindly provided by Prof. Rodríguez de Córdoba) for immunoblot detection. This serum produced a staining pattern identical to that previously observed (Fig. 2b, right) for both the C3-Del and C3-Sub variants (Fig. 3b). We next used a monoclonal antibody (mAb) recognizing the MG4–MG6 domains of the C3 β-chain [28]. Immunoblotting confirmed the presence of this epitope in both proC3-Del and proC3-Sub (Fig. 3c). Similarly, a mAb directed against the C-terminal region of C3 detected both variants (Fig. 3d).

In contrast, a mAb specific for the ANA/C3a domain failed to detect proC3-Del, while proC3-WT and proC3-Sub were readily detected (Fig. 3e). As this antibody recognizes its epitope under non-reducing conditions, the lack of reactivity toward proC3-Del may indicate an alteration of the folding or disulfide formation in this domain in the deletion variant.

Finally, to assess glycosylation, we performed lectin blotting using concanavalin A (ConA), this lectin has been used to enrich samples for C3 glycosylation analysis [31]. ConA staining was observed for proC3-WT and proC3-Sub, whereas proC3-Del exhibited a loss of binding, suggesting decreased or absent glycosylation (Fig. 3f, right). A parallel immunoblot for C3 was performed to confirm the presence of C3 (Fig. 3f, left). Deglycosylation of C3-WT and C3-Sub resulted in the loss of ConA binding, confirming that the observed signal was dependent on protein glycosylation (data not shown).

Collectively, these results indicate that, contrary to in silico predictions, the C3-Del variant is expressed intracellularly and retains several antigenic features of wild-type C3, consistent with the generation of a smaller protein product translated from a start site after the predicted frameshift, and lacking N-glycosylation. As expected, the C3-Sub variant is antigenically indistinguishable from C3-WT.

### C3-Del variant is exclusively located in the cytosol

Given the recent description of distinct forms of intracellular C3 within specific subcellular locations [19], the similarities between the cytosol-restricted C3 species and C3-Del, namely the lack of glycosylation and the absence of recognition by the anti-C3/C3a monoclonal antibody, we next performed cellular fractionation assays to define the intracellular localization of this variant. HepG2 C3-knockout (KO) cells were again used as a more physiologically relevant model.

**Fig. 4:**
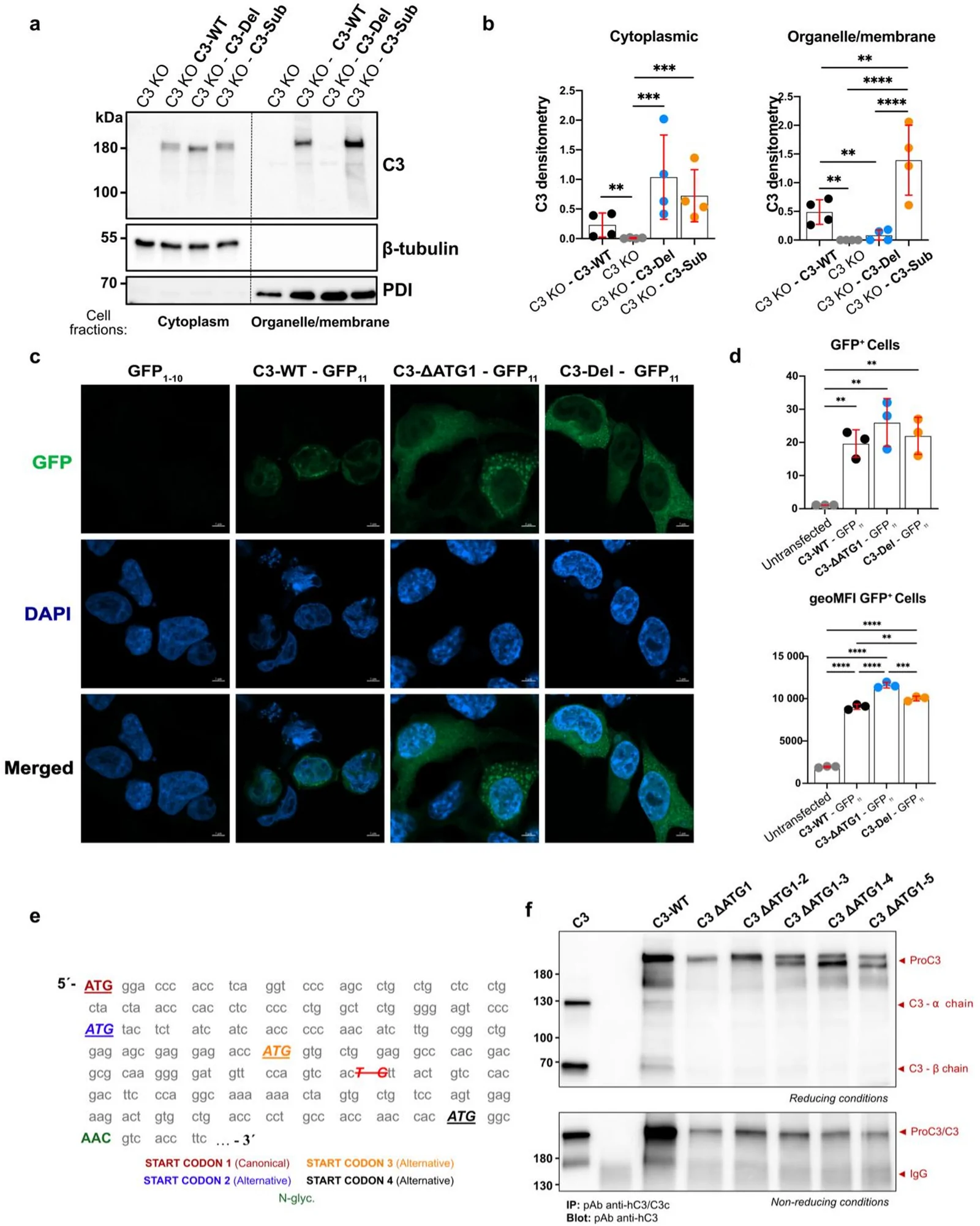
The C3-Del variant localizes exclusively to the cytosol, and its expression is rescued by an alternative start codon during translation. HepG2 C3 knockout (KO) cells were transfected with pcDNA3.1(+) plasmids encoding wild-type C3, C3-Del, or C3-Sub. Twenty-four hours post-transfection, cells were fractionated to obtain cytosolic and organelle/membrane-enriched fractions. ProC3 was immunoprecipitated from each fraction and analyzed by immunoblotting under reducing conditions (**a**), followed by densitometric quantification (**b**). (**c**) HEK293-sfGFP1-10 reporter cells were transfected with GFP11-tagged C3-WT, C3-Del, or C3-ΔATG1 constructs. GFP fluorescence was analyzed by confocal microscopy 24 h post-transfection. (**d**) Quantification of GFP-positive cells and geometric mean fluorescence intensity (geoMFI) by flow cytometry. (**e**) Schematic representation of the proposed fourth alternative start codon that may rescue C3-Del expression. N-glyc: N-glycosylation. (**f**) HEK 293 cells were transfected with different C3 constructs carrying mutations in the first five start codons. At 48 h post-transfection, C3 was immunoprecipitated and analyzed by immunoblotting. Data are presented as mean ± SD from 3 independent experiments. Statistical significance was determined using One-way ANOVA. *p = 0.01 − 0.05; **p = 0.001 − 0.0​1; *** *p* = 0.0001 − 0.001; ****p = < 0.0001

Under physiological conditions, the organelle/membrane fraction is enriched in canonical (secretory pathway-derived) C3, whereas the cytosolic fraction contains non-canonical cytosolic C3, along with minor amounts of retro-translocated canonical C3 [19]. Consistent with previous observations, C3-Sub exhibited a subcellular distribution similar to that of C3-WT (Fig. 4a-b), indicating that the c.1682G > A substitution does not impair intracellular trafficking.

In contrast, C3-Del was markedly enriched in the cytosolic fraction and was virtually absent from the organelle/membrane fraction (Fig. 4a-b). This distribution is consistent with earlier biochemical data and with the clinical phenotype of the patient, where no secreted C3 was detected in either cell supernatants or plasma.

We employed split-sfGFP technology to further confirm the cytosolic location of C3-Del (Fig. 4c). Briefly, the C3 constructs were fused to the GFP_11_ peptide. Expression of these constructs in the sfGFP_1−10_-enriched cytoplasmic compartment results in the reconstitution of GFP fluorescence and the generation of a bright fluorescent signal. Cells expressing C3-WT displayed a predominantly perinuclear GFP signal pattern, consistent with the canonical synthesis and secretion pathway of C3. A positive signal was observed since C3 can be retrotranslocated from the endoplasmic reticulum to the cytosol, as we have demonstrated in previous studies [19]. In contrast, cells expressing C3-ΔATG1, an exclusively cytosolic form of C3 used as a control, exhibited a diffuse GFP signal distributed throughout the cytoplasm. Similarly, cells expressing C3-Del showed a GFP localization pattern identical to that observed in the cytosolic C3-ΔATG1-expressing cells. No significant differences were observed in the percentage of GFP-positive cells (Fig. 4d), suggesting that the observed differences in GFP distribution were due to subcellular localization rather than differences in expression levels. Nevertheless, cells expressing C3-ΔATG1 or C3-Del exhibited a significantly higher geometric mean fluorescence intensity (geoMFI) for GFP compared with C3-WT-expressing cells (Fig. 4d), consistent with a higher proportion of C3-GFP_11_ in these cells being found in the cytosol.

Sequence analysis of C3 (Fig. 4e) revealed the presence of an ATG codon (+ 247–249) downstream of the c.168_169 deletion that could serve as an alternative start site, potentially functioning as a fourth translation initiation codon. Initiation from this site would generate an in-frame proC3 protein approximately 10 kDa smaller than wild-type proC3, closely matching the apparent molecular weight of the proC3-Del species observed experimentally.

To investigate this further, we generated C3 constructs carrying mutations in the first five canonical start codons (ATGs; M1I, M25I, M42I, M83I, and M164I) and evaluated their expression in HEK293 cells (Fig. 4f). As expected, mutation of ATG1-3 resulted in the expression of a smaller proC3 form. We next assessed the effect of additionally mutating ATG4 and ATG5. Surprisingly, C3 expression was still detected, with progressively smaller proC3 forms consistent with translation initiation from sites located downstream of ATG3. Notably, cells transfected with constructs in which ATG1-3 were mutated displayed two distinct proC3 bands, suggesting that translation may initiate at multiple downstream sites, potentially including non-canonical or near-cognate start codons (Supplementary Fig. 1).

Together, these findings support the conclusion that C3-Del expression is enabled by alternative translation initiation downstream of the deletion site, resulting in a non-canonical, cytosolic form of C3.

### C3-Sub is likely a loss-of-function variant that results in plasma C3 degradation

So far, we had not identified any defects in the synthesis, secretion or intracellular distribution of C3-Sub; however, circulating levels of this variant were previously reported to be markedly reduced [6]. Moreover, a case report described a patient with atypical hemolytic uremic syndrome (aHUS) harboring a mutation affecting the adjacent amino acid residue (p.S562L) to that altered in C3-Sub (p.G561D) [32]. Based on these observations, we next assessed the functional properties of recombinant C3-Sub.

**Fig. 5:**
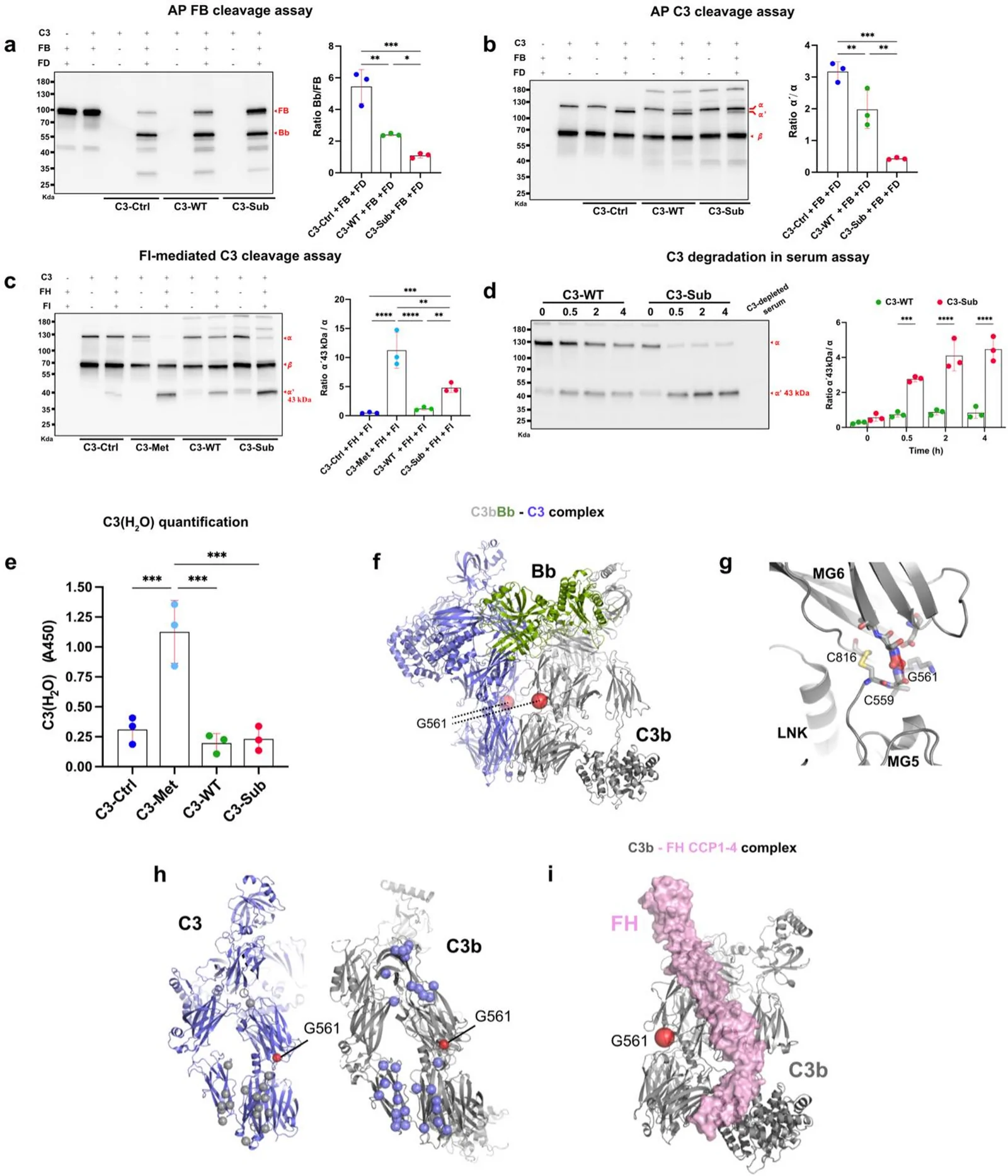
C3-Sub is a loss-of-function variant. Concentrated recombinant C3-WT and C3-Sub supernatants were incubated with purified factor B (FB) and factor D (FD). FB cleavage (**a**) and C3 activation (**b**) were assessed by immunoblotting. Factor H (FH) cofactor activity in the presence of factor I (FI) was also evaluated (**c**). Methylamine-treated C3 (C3-Met) served as a positive control. D). (**d**) Recombinant C3-WT or C3-Sub was incubated in 2% C3-depleted serum at 37 °C for the indicated time points, and C3 degradation was analyzed by Western blotting. Representative immunoblots and densitometric quantification of the ratio between the α′-43 kDa degradation fragment and the intact α-chain are shown. (**e**) Quantification of C3(H₂O) by ELISA. (**f**) Structural model of the alternative pathway C3 convertase (C3bBb) in complex with native C3 (PDB ID: 9U62), highlighting in red the location of the residue Gly561 (C3b in gray, Bb in green, native C3 in blue). (**g**) Region surrounding Gly561; this is positioned adjacent to the conserved intrachain disulfide bond (yellow) formed between Cys559 in the C3 β-chain and Cys816 in the C3 α-chain. (**h**) Interaction surface between C3 and C3b in the AP C3 convertase; grey spheres on C3 are those residues in contact with C3b, and the blue spheres on C3b are those residues that are in contact with C3. The G561 residue is shown in red. (**i**) Structural model of C3b (gray) in complex with factor H CCP1–4 (pink, PDB ID: 2WII), showing the position of Gly561 in red near the interaction surface between C3b and factor H. Data are presented as mean ± SD from 3 independent experiments. Statistical significance was determined using One-way ANOVA. *p = 0.01 − 0.05; **p = 0.001 − 0.0​1; *** *p* = 0.0001 − 0.001; ****p = < 0.0001

We first performed an alternative pathway (AP) C3 convertase assembly assay. In the presence of factor B (FB) and factor D (FD), C3-Sub induced a significant reduction (approximately 50%) in the proteolytic cleavage of FB into Ba and Bb compared with C3-WT (Fig. 5a). Consistently, a marked decrease (approximately 75%) in C3 activation to C3a and C3b was also observed (Fig. 5b). These results indicate that C3-Sub has a diminished capacity to efficiently participate in AP C3 convertase assembly.

Immunoblot analysis revealed no evidence of protein degradation, and the apparent molecular weights of the C3 α- and β-chains were as expected. Therefore, we next evaluated the functional state of C3-Sub using a factor H (FH) cofactor activity assay (Fig. 5c). When incubated with FH and factor I (FI), C3-Sub was cleaved to iC3b at a significantly higher rate than C3-WT (approximately 5-fold), and in a manner comparable to C3-Met.

Given the results obtained thus far, we hypothesized that C3-Sub may exhibit an increased tick-over rate or preferentially adopt a C3(H₂O)-like conformation. To investigate this, recombinant C3-WT or C3-Sub was added to diluted C3-depleted serum, and C3 degradation was evaluated by quantifying the ratio of the α′-43 kDa degradation fragment to the intact α-chain at different time points. Notably, a significant increase in C3 degradation was observed for C3-Sub at all analyzed time points (Fig. 5d). In addition, the levels of C3(H₂O) were quantified for both C3-WT and C3-Sub, using methylamine-treated C3 (C3-Met) as a positive control. No significant differences were observed between C3-WT and C3-Sub (Fig. 5e), indicating that the mutation present in C3-Sub does not promote adoption of a C3(H₂O)-like conformation.

To further investigate the potential functional consequences of the mutation, molecular modelling was performed to map the location of the mutated residue, Gly561, onto the cryo-EM structure of the alternative pathway C3 convertase (C3bBb) in complex with native C3 (Fig. 5) and the crystal structure of C3b in complex with factor H CCP1-4 (Fig. 5). Gly561 was found to localize in proximity to the C3-C3b interaction interface within the C3bBb-C3 complex (Fig. 5h). Similarly, the mutated residue was located near the interaction surface between C3b and factor H CCP1-4 (Fig. 5i), suggesting that the mutation may influence protein-protein interactions involved in complement activation and regulation.

Together, these findings indicate that the C3 c.1682G > A (p.G561D) variant represents a loss-of-function mutation that enhances spontaneous C3 degradation in plasma by FH and FI.

## Discussion

The description of the intracellular complement or complosome [15, 33]​ has expanded C3 biology knowledge by revealing non-canonical functions that are largely independent of its extracellular immune role. These observations raise an important question: how do pathogenic C3 variants affect intracellular C3 biology? Given the increasing recognition of intracellular complement biology, this represents a significant gap in our understanding. Primary deficiency of complement C3 cases offer a unique opportunity to explore this question. In the present study, we therefore investigated, for the first time, the intracellular behavior of two previously reported C3 deficiency-causing variants.

The *C3 c.168_169del* variant was predicted to introduce a frameshift resulting in the production of a truncated short peptide due to a premature termination codon [6]. Unexpectedly, we detected intracellular proC3 in cells transfected with the corresponding construct. Thus, our findings demonstrate that extracellular C3 deficiency cannot automatically be interpreted as complete cellular C3 deficiency.

Our data support​ the hypothesis that while *C3 c.168_169del* variant abolished secretion and plasma availability, intracellular C3 expression persisted. This is likely due to translation being re-initiated at a downstream start codon, with the mutant predicted truncated product (Fig. 1b) from the canonical start site acting as an upstream open reading frame [34], resulting in loss of the leader peptide and consequent retention in the cytosol. Although the precise translation initiation site was not experimentally validated, the observed molecular weight, cytosolic localization, and biochemical properties strongly support initiation downstream of the c.168_169del mutation.

Biochemical characterization of C3-Del revealed preserved antigenicity with several monoclonal antibodies and polyclonal sera, comparable to C3-WT. However, two notable differences were observed: absence of recognition by a monoclonal antibody targeting the ANA/C3a domain and lack of ConA binding, consistent with altered, decreased, or absent mannose-rich glycan residues on the glycosylation sites of C3. In addition, the concentration of C3-Del in cell lysates was significantly lower than that of C3-WT. We speculate that this difference may reflect less efficient translation initiation from the alternative start codon. However, we cannot exclude the possibility that conformational differences between C3-Del and C3-WT may reduce recognition of C3-Del by the polyclonal antiserum used in this study, which directly affects antibody recognition-based assays.

The cytosolic forms of C3 are directly synthesized within this compartment. Given the reducing environment of the cytosol, we hypothesized that correct disulfide bond formation may be impaired, potentially resulting in conformational differences between cytosolic C3 and the canonical/secreted form. This may​​ be particularly relevant for the C3 ANA domain, which contains three disulfide bonds. The absence or altered formation of these bonds in the cytosol could affect the conformation of the ANA domain and, consequently, impair recognition by the antibody used in this study. Consistent with this possibility, this antibody recognizes C3 under non-reducing immunoblotting conditions, when disulfide bonds remain intact.

To further support this hypothesis, the cytosolic localization of C3-Del was confirmed by cellular fractionation experiments and split-GFP assays. These analyses demonstrated that C3-Del behaves similarly to the cytosol-restricted C3-ΔATG1 construct, exhibiting comparable expression levels and fluorescence patterns following transfection, in contrast to C3-WT. Collectively, these findings confirm C3-Del as a cytosol-exclusive form of C3.

These findings are consistent with our previous work demonstrating that C3 mRNA contains two alternative downstream start codons that generate a cytosolic C3 isoform [19]. Importantly, we have previously demonstrated functions of this cytosolic form of C3, which rescues detrimental phenotypes of C3-KO cells in the absence of extracellular C3 [20, 21].

The presence of a functional start codon downstream of the deletion site suggests the existence of additional non-canonical translation initiation sites within the C3 transcript. We propose that a third non-canonical start codon is responsible for the generation of cytosol-exclusive C3 isoforms (start codon 4 in Fig. 4e). Likewise, our findings point to the existence of multiple translation initiation sites for C3. When the first five canonical ATG start codons were sequentially mutated, progressively smaller proC3 forms were produced. Interestingly, following mutation of ATG1-3, additional bands corresponding to higher-molecular-weight proC3 forms were also detected. Given that the canonical initiation sites were disrupted, we hypothesize that this larger proC3 may result from translation initiation at non- or near-cognate start codons [35]. Notably, such higher-molecular-weight proC3 forms were not detected for C3-Del. One possible explanation is that some of these alternative initiation sites are located upstream of the C3-Del deletion/frameshift locus (Supplementary Fig. 1). Translation initiated at these sites would therefore generate products affected by the deletion, potentially resulting in truncated or unstable proteins that are not detectable by immunoblotting. In contrast, alternative initiation sites located downstream of the deletion would remain available for translation reinitiation after the premature frame-shifted STOP codon [36], accounting for the smaller C3-Del protein form observed. Recent studies have shown that the use of non-AUG start codons is not uncommon in human cells and may represent a versatile mechanism for regulating protein expression and expanding proteomic diversity [35, 37–39]. Together, our findings suggest that the translational regulation of canonical and non-canonical C3 forms is more complex and diverse than previously recognized, potentially reflecting the biological relevance of generating distinct C3 isoforms.

The presence of multiple potential alternative translation initiation sites within the C3 gene may therefore suggest evolutionary selection for isoform diversity. Alternative translation initiation through mechanisms such as ribosomal leaky scanning or re-initiation has been described for other proteins [40–42], and it is suggested that this may expand proteomic diversity from a single transcript [43].

Notably, C3 is an ancient protein that emerged more than 800 million years ago [44] and is a member of the thioester-containing protein (TEP) family [45]. TEP family members are predominantly secreted proteins involved in extracellular immune regulation and protease inhibition [45–47], with the complement cascade representing one of the most prominent examples. However, the evolutionary emergence of intracellular C3 functions remains unresolved. In this context, the preservation of multiple translation initiation sites within the C3 gene may reflect evolutionary pressure favoring functional diversification, allowing canonical extracellular immune functions to coexist with intracellular roles in cellular metabolism and homeostasis [18, 48].

In contrast to C3-Del, the C3-Sub (p.G561D) variant showed no detectable differences in intracellular synthesis, secretion, or antigenicity compared with C3-WT. However, patients harboring this variant exhibit profound plasma C3 depletion. Functional analyses revealed impaired assembly of the fluid-phase alternative pathway C3 convertase, as evidenced by reduced proteolysis of C3 and factor B. Additionally, we observed increased susceptibility to factor I-mediated proteolysis in the presence of factor H, although not to the extent seen with C3(H_2_O).

Our findings suggest that C3-Sub may preferentially adopt a conformation or form an intermediate that displays increased FI-mediated degradation, leading to enhanced degradation without completely abolishing factor B binding. Our data indicate that native C3-Sub does not assume a conformation in which the ANA domain is exposed, as observed for C3(H_2_O) or C3-Met. However, the C3 activation intermediate, C3*, adopts a conformation that is markedly distinct from those of both native C3 and C3-Met [49], highlighting the substantial conformational flexibility of C3 and supporting the possibility that C3-Sub may adopt a distinct structural state. This finding supports the hypothesis that C3-Sub adopts a distinct conformational state that renders it more susceptible to factor I-mediated proteolytic cleavage.

Moreover, incubation of C3-Sub in C3-depleted serum resulted in increased C3 degradation, potentially due to the destabilization of the MG-ring arising from the steric hindrance imposed by the Asp side chain substituting for Gly561. Notably, Gly561 is located near the intrachain disulfide formed between Cys559 in the C3 β-chain and the Cys816 in the C3 α-chain (Fig. 5g) that links the MG6 domain to the MG5-MG6 linker. Such structural perturbation may impair the interaction between MG2 and MG6 domains of C3-Sub and the CCP2 domain of FB, a contact that is essential for the proconvertase C3bB formation required for FD-mediated cleavage of C3bB into the active C3 convertase (C3bBb) [50]. This mechanism may explain the severe plasma depletion observed in patients: rather than impaired production, C3-Sub appears to undergo accelerated consumption. Notably, intracellular levels were comparable to C3-WT, further supporting compartment-specific effects.

To our knowledge, p.G561D represents the first reported case in which a C3 loss-of-function variant paradoxically results in plasma C3 deficiency due to increased degradation despite normal secretion. These findings have diagnostic implications, as the p.G561D variant may be more accurately classified as causing secondary C3 deficiency due to consumption rather than primary deficiency due to impaired synthesis. In addition, although the C3-Del mutation leads to primary serum deficiency, it is still expressed intracellularly and can be expected to maintain intracellular functions, thus explaining the positive staining of C3 in patient-derived cells despite the lack of extracellular C3 [6].

Collectively, our data demonstrate that two previously described C3 deficiency-associated variants remain stable in the intracellular compartment and exhibit distinct intracellular behaviors. These findings underscore the importance of experimentally characterizing pathogenic variants beyond in-silico predictions, which may oversimplify complex biological processes or misclassify variants, as has been described for other complement components such as factor H [51]. These new data not only improve the databases for bioinformatic prediction software but also provide precious information on basic biological processes involving C3.

Nevertheless, it is important to acknowledge that our evidence was obtained primarily through in vitro experimentation. These findings should therefore be complemented by in vivo studies and analyses of patient-derived samples. In the present study, we were unable to obtain additional cellular samples from the patient carrying the C3-Del variant or fresh serum samples from the patient carrying the C3-Sub variant.

These limitations highlight the need to update current diagnostic approaches to better assess the effects of pathogenic C3 variants, as well as variants of uncertain significance, on the intracellular biology of C3. Future studies should establish standardized protocols defining the most appropriate sample types for evaluating intracellular C3 and develop patient-derived cellular models that enable functional characterization of these variants in a more physiologically relevant context.

The functional independence of intracellular and extracellular C3 pools is highlighted by the divergent biochemical properties and compartment-specific consequences of the variants studied here. These observations emphasize the need to integrate intracellular complement functions into our understanding of complement-related diseases. Such integration may refine diagnostic classification and potentially identify novel therapeutic targets.

In conclusion, our findings reveal compartment-specific effects of pathogenic C3 variants and reinforce the concept that intracellular and extracellular complement systems represent functionally independent yet interconnected compartments. Recognizing this duality will be essential for advancing both mechanistic insight and clinical management of complement-mediated disorders.

## Supplementary Information

Below is the link to the electronic supplementary material.

Supplementary Material 1

## Data Availability

No large datasets of public interest were generated during the current study. Any relevant experimental data are available from the corresponding author on reasonable request.
