# Supplementary material for: Genetic C3 variants reveal functional independence between extracellular and intracellular forms of complement C3
Source: Cell Mol Life Sci. 2026 Nov 3;83(1):409. doi: 10.1007/s00018-026-06517-2 (PMC13739894; doi:10.1007/s00018-026-06517-2)
Supplement: Supplementary file 1 [file 18_2026_6517_MOESM1_ESM.pdf]

Supplementary Information

Genetic C3 Variants Reveal Functional Independence between Extracellular and Intracellular Forms of Complement C3

Daniel Chacon-Arguedas[1] (0000-0002-1968-8381), Ben C. King[1] (0000-0001-6776-8741), Ana V. Marin[2] (0000-0002-7541-3729), Agustin Tortajada[2] (0000-0002-2131-2594), Gregers Rom Andersen[3] (0000-0001-6292-3319), Jose R. Regueiro[2‡] (0000-0001-8442-7762) & Anna M. Blom[1†‡] (0000-0002-1348-1734).

1. Lund University, Department of Translational Medicine, Malmö, Sweden.
2. Complutense University School of Medicine and Institute of Health Research 12 de Octubre (imas12), Department of Immunology, Ophthalmology and ENT, Madrid, Spain.
3. Aarhus University, Department of Molecular Biology and Genetics, Aarhus, Denmark.

†Corresponding Author: anna.blom@med.lu.se

‡ These authors contributed equally as senior authors.

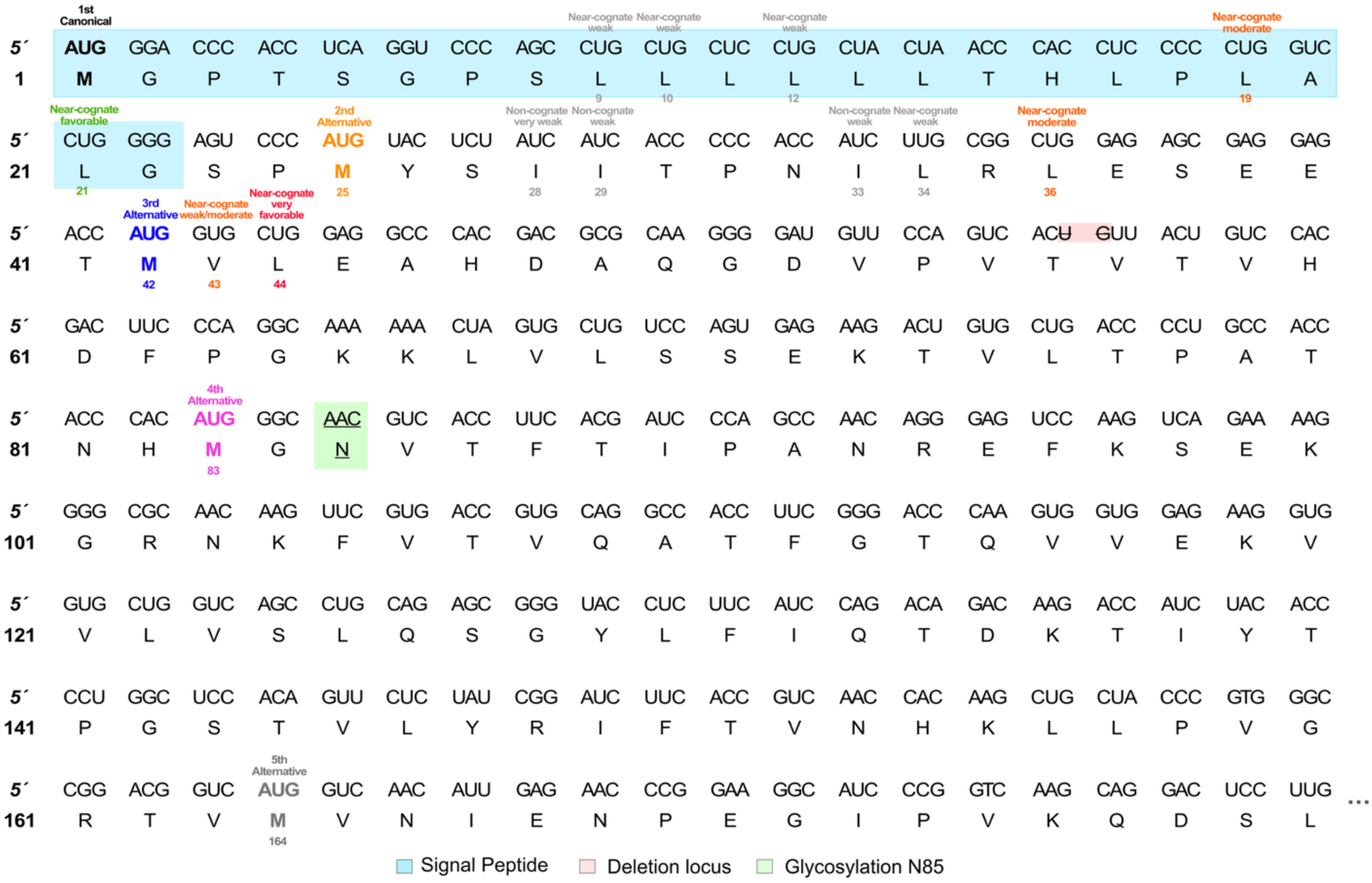

Supplementary Figure 1. Alternative translation initiation sites within the 5′ region of the human C3 coding sequence. Schematic representation of the first 164 codons of C3, showing the canonical translation initiation codon (ATG1; M1) and the four downstream alternative ATG start codons (ATG2-5; M25, M42, M83, and M164). Potential near-cognate translation initiation codons upstream of ATG4 are indicated and classified according to their predicted initiation strength (very favorable, favorable, moderate, or weak). The sequence encoding the canonical C3 signal peptide is highlighted in blue, the region corresponding to the C3-Del deletion locus in red, and the N-glycosylation site at N85 in green. Nucleotide and amino acid sequences are shown above and below each codon, respectively.
